# An Anti-hTNF-α Variable New Antigen Receptor Format Demonstrates Superior *in vivo* Preclinical Efficacy to Humira® in a Transgenic Mouse Autoimmune Polyarthritis Disease Model

**DOI:** 10.3389/fimmu.2019.00526

**Published:** 2019-03-22

**Authors:** Obinna C. Ubah, John Steven, Andrew J. Porter, Caroline J. Barelle

**Affiliations:** ^1^Elasmogen Ltd, Aberdeen, United Kingdom; ^2^Scottish Biologics Facility, School of Medical Sciences, University of Aberdeen, Aberdeen, United Kingdom

**Keywords:** variable new antigen receptors (VNARs), TNF-alpha, autoimmune disease, rheumatoid arthritis, anti-TNF biologics, shark IgNAR, chronic inflammation

## Abstract

Tumor necrosis factor-alpha (TNF-α), an established pro-inflammatory cytokine plays a central role in the induction and progression of several chronic inflammatory and autoimmune diseases. Targeting TNF-α as a treatment modality has shown tremendous success, however there are several limitations associated with the current anti-TNF-α biologic drugs including: immunogenicity, life-threatening infections, resistance to treatment, complexity of manufacture and cost of treatment. Here, we report the *in vivo* efficacy of novel anti-TNF-α formats generated from molecular engineering of variable new antigen receptors (VNARs), originally derived from the immune system of an immunized nurse shark. Two anti-TNF-α VNAR formats, a tandem multivalent trimer, D1-BA11-C4 and an Fc-fused quadrivalent D1-Fc-C4 (Quad-X™) construct were tested in a clinically relevant, preclinical mouse efficacy model of polyarthritis (Tg197) and compared to the commercial anti-TNF-α “best in class” therapy, Adalimumab (Humira®). Both VNAR formats bind and neutralize TNF-α through an epitope that appears to be different from those recognized by other anti-TNF biologics used clinically. All doses of Quad-X™, from 0.5 to 30 mg/kg, significantly blocked the development of polyarthritis. At 0.5 mg/kg Quad-X™, the arthritis score was improved by 76% and the histopathology score by 63%. At 3 mg/kg Quad-X™, control of disease was almost complete at 90% (arthritis) and 88% (histopathology). In marked contrast, 1 mg/kg Humira® saw profound disease breakthrough with scores of 39 and 16% respectively, increasing to a respectable 82 and 86% inhibition at 10 mg/kg Humira®. We have previously reported the superior potency of anti-TNF-α VNARs *in vitro* and in these studies translate this superiority into an *in vivo* setting and demonstrate the potential of VNAR formats to meet the requirements of next-generation anti-TNF-α therapies.

## Introduction

Tumor necrosis factor-alpha (TNF-α) is a pleiotropic cytokine with beneficial functions in immune regulation and host defense, but when expressed at high levels, is also implicated in a number of human diseases. TNF-α is a critical mediator of the autoimmune process, playing a key role in several inflammatory diseases ranging from rheumatoid arthritis, inflammatory bowel diseases, psoriasis, and uveitis. Rheumatoid arthritis (RA) is a common, chronic inflammatory disorder that causes progressive articular destruction and is associated with comorbidities in vascular, metabolic, bone and even the psychological well-being of sufferers (1, 2). Affecting about 1% of the global population, RA patients are faced with functional disability, pain, joint damage, a reduced quality of life, and even premature mortality, with a corresponding and significant negative economic impact on both the patients and government health systems struggling to treat the disease (3, 4). The management of many autoimmune mediated diseases has significantly improved in the last decade because a better understanding of disease mechanisms has guided the development of new and potent therapies. The mainstay treatment for steroid-refractory RA is *via* the targeted neutralization/blocking of the TNF-α signaling pathway with anti-TNF-α or anti-TNF receptor biologics. The monoclonal antibody, Adalimumab (Humira®) is the most widely used and commercially successful TNF-α antagonist monoclonal antibody (5–7). However, there still remain some significant limitations with this treatment approach. Approximately 50% of patients fail to see their disease properly controlled with the existing battery of anti-TNF biologics. A failure to respond to treatment or the development of resistance to therapy (through immunogenicity or other mechanisms), the use of progressively higher doses (10–30 mg/kg) with prolonged therapy windows, concomitant, and often significant systemic side-effects (infection, heart problems, malignancy) are just few of the limitations associated with the current anti-TNF biologics in the clinic (8–16). Some of these limitations are in part also related to the molecular mass and structural complexity of mAbs which can increase manufacturing costs and clinically limit routes of administration.

The Variable New Antigen Receptors (VNARs) are the smallest (11 kDa) naturally occurring independent binding domains in the vertebrate kingdom (17–19). They play an integral role in the adaptive immune system in cartilaginous fish and although they are structurally similar to mammalian heavy and light variable chains it has been well-documented that they arose from a distinct evolutionary lineage from Immunoglobulins (20). A lack of CDR2 and the addition of two loops of diversity (HV2 and 4) as well as low percentage sequence homology exemplifies this distinction. Their characteristic protruding paratopes, often referred to as “canyon-binders,” predisposes these domains to access and bind epitopes not normally available to conventional biologics and encourages the selection of highly potent neutralizers specific for enzyme and/or receptor targets (21–23). We have previously demonstrated the ease with which reformatting of VNAR domains can be achieved delivering: multivalent, bi/tri-specific constructs, and serum half-life extension through molecular fusion to an anti-human serum albumin (HSA) VNAR scaffold, NDure™ or *via* a more traditional route using IgG (mouse or human) Fc domains (24–26). These highly stable constructs can be expressed cost-efficiently and at scale in non-mammalian systems. This menu of format options permits tailored selection of drug modalities optimized for systemic, site-specific, or topical administration of VNAR candidates.

Here, we have demonstrated that an anti-hTNF-α VNAR construct (Quad-X™) previously reported as having potent *in vitro* activity (24) retains this superiority to the commercially available blockbuster anti-TNF-α, Adalimumab (Humira®) *in vivo* and is capable of preventing the development of spontaneous polyarthritis in a transgenic mouse model of arthritis at doses below 3 mg/kg. Also, and for the first time, we have shown that sustained *in vivo* efficacy is possible from a non-Fc fused anti-HSA half-life extended linear VNAR construct following systemic administration in the same RA disease model.

## Materials and Methods

### Anti-hTNF-α VNAR Domain Reformatting, Expression and Purification

**Figure 1:**
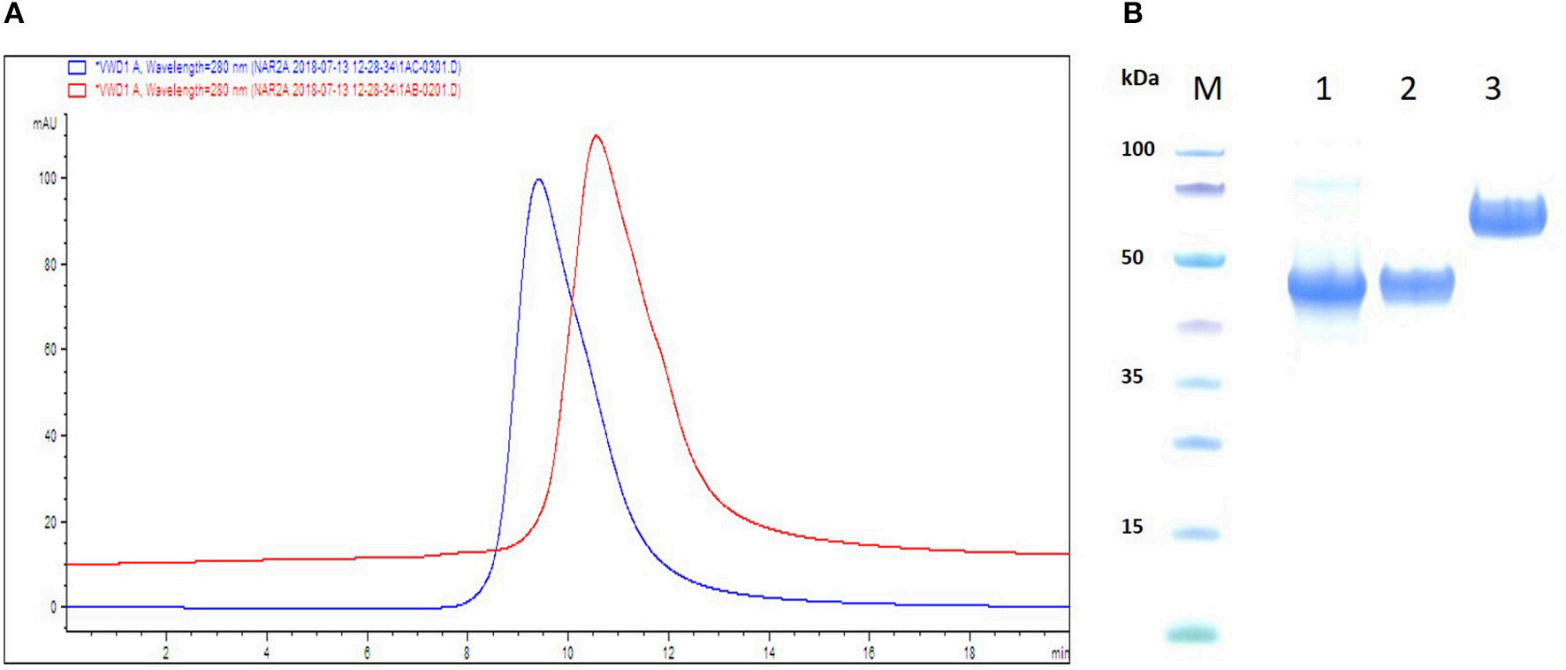
Schematic ribbon illustration, analytical SEC and SDS-PAGE profile of purified VNAR test articles. **(A)** SEC traces are shown as overlaid and offset to aid comparison. The chromatograms confirm no evidence of aggregation or impurities in the samples. Red trace—D1-BA11-C4, Blue trace—Quad-X™. **(B)** SDS-PAGE and Coomassie blue staining of 5 μg of dithiothreitol-treated VNAR constructs. M, molecular weight marker; Lane 1, VNAR D1-BA11-C4; Lane 2, VNAR-Fc standard control (≈ 45 kDa); Lane 3, D1-Fc-C4 (Quad-X™) Ubah et al. (24).

The rational design and reformatting of the two anti-hTNF-α VNAR test articles (D1-BA11-C4 and D1-Fc-C4 (Quad-X™) has been described in a recent publication (24). D1-BA11-C4 is a 40 kDa trivalent, bi-specific, bi-paratopic VNAR construct targeting hTNF-α *via* the D1 and C4 domains molecularly fused with a validated, clinical-study ready, serum half-life extension humanized VNAR (soloMER™) domain, BA11 (NDure™) targeting serum albumin (Figure 1). The VNAR domains in the trimer construct are separated by flexible (Gly_4_Ser)_4_ linkers and contain a C-terminally fused 6x Histidine tag to facilitate purification. The Quad-X™ construct (Figure 1), a 105 kDa fusion molecule was designed with two varying GlySer linker lengths; a short (Gly_4_Ser)_2_ fusing the first anti-hTNF-α VNAR domain (D1) to the hinge of a wild-type human IgG1 Fc region, and a longer (Gly_4_Ser)_4_ linker fused to the C-terminal of an Fc CH-3 linking to a second anti-hTNF-α VNAR domain (C4). Purification of this construct is *via* the human IgG1 Fc, using Protein-A affinity chromatography. Both constructs were expressed in suspension-adapted CHO K1 cells.

### Cross-Reactivity Binding ELISA and Cytokine Neutralization Assay

ELISA plates were coated with human, mouse, rat, dog, cynomolgus, rabbit, pig TNF-α and human TNF-β at 1 μg/ml concentration in PBS. Plates were incubated for 1 h at 37°C, then blocked with 2% Milk-PBS. Plates were washed 3x with PBS and 0.001% (v/v) Tween-PBS (PBST). Anti-TNF-α VNAR D1 and C4, VHH TNF30 and Humira® were added to designated ELISA wells at a 5 μg/ml top concentration with 2-fold serial dilution. Plates were incubated at room temperature for 1 h, washed as described above. TNF-α binding activity was detected using appropriate secondary antibodies; VNARs D1 and C4, and VHH TNF30 were detected using anti-poly-histidine-HRP, and Humira® was detected using an HRP-conjugated anti-human IgG1 Fc antibody.

An *in vitro* TNF-α neutralization assay in a mouse fibrosarcoma (L929) cell line was conducted as described in Ubah et al. (24). LD_50_ doses for the TNF-α species, as determined through cytokine titration in the same L929 cells cytotoxicity assay, are as follows; human TNF-α (0.3 ng/ml), mouse TNFα (0.05 ng/ml), cynomolgus TNF-α (0.15 ng/ml), pig TNF-α (100 ng/ml), canine TNF-α (0.5 ng/ml), and human TNF-β (0.5 ng/ml). A neutralization screen was not performed when all the test anti-TNF-α domains (VNARs, VHH and Humira®) failed to bind a target species TNF-α, e.g., rat and rabbit TNF-α.

### Analytical SEC & SDS-PAGE Profile of Recombinant Anti-hTNF-α VNAR Domains

Aggregation propensity of the reformatted VNAR test articles was analyzed using analytical size-exclusion chromatography (SEC) and an Agilent 1200 series HPLC system with a ZORBAX GF 250 9.4 × 250 mm 4 μm column containing phosphate buffered saline pH 7.4 as the mobile phase. VNAR D1-BA11-C4 and Quad-X™ were loaded onto columns at 6 mg/ml from stock concentrations of 40 mg/ml. Purity of the test VNAR protein samples was also assessed by SDS-PAGE electrophoresis of the reduced VNAR protein samples in a MES buffer system (Invitrogen).

### Mice

C57BL/6 and Tg197 mice were used for the maximum tolerated dose (MTD) and polyarthritis prevention studies, respectively and were provided by Biomedcode Hellas SA, Greece. All reported animal studies were conducted at the Biomedcode SA test facility. All procedures conformed to the Presidential Decree No 56/2013 Government Gazette No A' 106 applicable in Greece (EEC Directive 2010/63/ECC) and were approved by the directorate of Agricultural and Veterinary Policy (DAVP) of the Attica Region. Approved license Protocol No. 3798/3-07-2017.

### Treatments

Anti-human TNF-α VNARs, D1-BA11-C4 and D1-Fc-C4 (Quad-X™) were produced recombinantly in suspension-adapted CHO K1 cells by Evitria AG, Switzerland. *In vitro* functional activity of these expressed proteins was confirmed (24), and “safe” endotoxin levels determined using a Pierce™ LAL Chromogenic Endotoxin Quantitation Kit (Product no. 88282) (Data not shown). Humira®, a gold standard anti-hTNF-α human monoclonal antibody (human IgG1 Fc) was locally purchased by Biomedcode SA (Lot/batch No. 74270XH07, Expiry date 01/2019). D-PBS was used as the stock solution diluent and the vehicle control was again purchased locally by Biomedcode SA (Lot/batch no. 1892388, expiry date 06/2020).

### Maximum Tolerated Dose (MTD) in C57BL/6 Mice

Anti-hTNF-α Quad-X™ was evaluated for safety and tolerability in healthy mice. A pool of 6-week-old C57BL/6 mice were allocated to 4 groups (G1-G4), each consisting of 10 gender and age matched mice, based on an equal mean group body weight distribution. On days 0 and 3, animals received subcutaneous administration of either vehicle (PBS, G1), 3 mg/kg (G2), 10 mg/kg (G3), or 30 mg/kg (G4) of anti-hTNF-α Quad-X™. Treated animals were monitored daily for 7 consecutive days to assess any possible adverse effects by recording animal viability, overall appearance, body function, behavior, and body weight. Water and food intake were also assessed at 2 different time points during the study. At the end of the clinical monitoring period, mice were sacrificed, and gross necropsy determined. The heart, liver, spleen and kidneys from all experimental animals were collected and weighed to determine organ to body weight ratios.

### Polyarthritis Prevention in the Tg197 Mouse Model

The transgenic Tg197 mouse model was used to investigate the efficacy of the anti-hTNF-α VNAR D1-BA11-C4 and Quad-X™ formats and compared to the positive control article Humira®, as described in Keffer et al. (27). The Tg197 mice carry a human TNF transgene, which has been modified to include a 3′-untranslated region sequence from a beta-globin gene, thereby allowing deregulated human TNF gene expression. In mice containing this genetic background, chronic polyarthritis incidence is 100% by week 4 of age. In this therapeutic model, treatment is aimed at preventing establishment of disease by blocking the biological activity of the deregulated hTNF-α expression.

This study was conducted using two independent experiments. In the first, 5 groups of 8 sex and age matched mice (4♂/4♀) were assigned to one of five treatment regimens. PBS treatment, three different dosing regimens of VNAR Quad-X™ and one dosing regimen of Humira®. Doses of 3, 10, 30 mg/kg (Quad-X™), and 10 mg/kg (Humira®) were administered subcutaneously twice weekly, starting at week 3 after birth until 10 weeks of age.

In the second study, 7 groups of 8 mice each (4♂/4♀) were assigned to one of seven treatments regimens of twice weekly subcutaneous administration. VNAR Quad-X™ was dosed at 0.5, 1, and 3 mg/kg; VNAR D1-BA11-C4 at 30 mg/kg; and Humira® at 1 and 3 mg/kg.

An additional group of untreated mice for baseline histopathological status (3-week-old control mice) were sacrificed prior to the first dose administration. An *in vivo* arthritis scoring system (28) was applied based on the macroscopic changes observed in joint morphology on both ankle joints of the hind limbs. An arthritis score (AS) was recorded weekly on both ankle joints, and average scores were calculated. All mice were sacrificed 48 h after administration of the last dose (week 10 of age), and two ankle joints of each animal were collected for histopathological evaluation.

#### Histopathological Evaluation

Ankle joints were fixed in 4% aqueous formaldehyde solution overnight at room temperature, demineralised in EDTA decalcification solution (13% EDTA in 0.1 M sodium phosphate buffer) at room temperature for 30 days and placed in PBS at 4°C until required for further processing. Samples were paraffin embedded in the sagittal plane, and paraffin blocks sectioned. Blocks were stained with Haematoxylin and Eosin (H&E) staining and evaluated by light microscopy for histopathological hallmarks of arthritis in accordance with a standard histopathology scoring system (29, 30). The evaluation process was performed in a blinded fashion based only on a slide number and not the sample ID. Only representative images were acquired (25x magnification), and group means were calculated from the individual highest scores per joint in each mouse in each specific group.

### Preclinical Study Amendments and Deviations

In the MTD study, during the necropsy procedure and organ collection weighing, the right kidneys of two mice (one in 10 mg/kg Quad-X™ and the other in the 30 mg/kg Quad-X™ treatment group) were not recovered, therefore no weight of the right kidney was recorded. There were no other study amendments or deviations, and no deaths were reported in both the MTD and Efficacy Preclinical studies.

### Statistical Analysis

Statistical analyses were performed using GraphPad Prism 6 software. Statistical tests applied to each data set are specified in each figure legend. Statistically significant differences were noted as: ^*^p < 0.05, ^**^p < 0.01, ^***^p < 0.001, ^****^p < 0.0001.

## Results

### SEC & SDS-PAGE Analysis of VNAR Test Articles

VNAR test articles were expressed, purified and assessed for function in both binding ELISA and classical L929 fibrosarcoma cells—hTNF-α neutralization assay (24). Analytical SEC was performed to determine the presence of any aggregation in concentrated (40 mg/ml) reformatted VNAR protein samples prior to their *in vivo* use. Both VNAR D1-BA11-C4 and Quad-X™ eluted as single peaks and showed no signs of aggregation or impurities. In addition, SDS-PAGE analysis of 5 μg VNAR D1-BA11-C4 and Quad-X™ confirmed the SEC analysis with no evidence of degradation or impurities observed (Figures 1A,B).

### Species Cross-Reactivity Characterization of Lead Anti-TNF VNAR Domains

The lead individual anti-TNF-α VNAR domains (D1 and C4) showed positive reactivity to only human, dog and cynomolgus TNF-α. There was no binding seen with any of these VNAR domains to rat, mouse, rabbit, pig TNF-α or closely related human TNF-β (Table 1A). These binding recognition and neutralization profiles clearly separated these two VNAR binding domains from the profile seen with Humira® and the VHH TNF30. Humira® binds and neutralizes human, dog, cynomolgus and mouse TNF-α, while VHH TNF30, in addition to binding and neutralizing human, dog, cynomolgus and pig TNF-α, binds human TNF-β.

**Table 1A:** Cross-reactivity profile of anti-hTNF-alpha VNAR lead constructs compared to commercially available anti-hTNF-alpha mAbs and a pre-clinical VHH TNF30 domain. **(A)**. Binding (B) and Neutralization (N) data obtained in our laboratory.

	**Human** ** (bind/neutralize)** ** B/N**	**Dog** ** (B/N)**	**Cynomolgus** ** (B/N)**	**Rat** ** (B/N)**	**Mouse** ** (B/N)**	**Rabbit** ** (B/N)**	**Pig** ** (B/N)**	**Human TNF-β** ** (B/N)**
Lead anti-hTNF-α VNARs (D1 & C4)	+++/+++	+++/+++	+++/+++	–/–	–/–	–/–	–/–	–/–
TNF30 (VHH)	+++/+++	+++/+++	+++/+++	–/–	–/–	–/–	+/+	++/–
Adalimumab (Humira®)	+++/+++	+++/+++	+++/+++	–/–	++/++	–/–	–/–	–/–

***+++** denotes strong binding/neutralization activity, **++** moderate; **+** very weak activity and**—**denotes no binding/neutralization activity observed. Binding (B) and Neutralization (N). TNF30 is an anti-TNF-α Nanobody™ previously reported in Beirnaert (31)*.

Clinically available anti-TNF-α therapies including Infliximab (Remicade®) a mouse-human IgG1-kappa, Certolizumab (Cimzia®) a PEGylated Fab fragment of a humanized mAb, Golimumab (Simponi®) a fully human IgG1-kappa mAb, and Etanercept (Enbrel®) a fusion protein of human TNF receptor 2, all show restricted TNF-species cross-reactivity with recognition profiles different from those determined for the anti-TNF-α VNARs. Infliximab binds and neutralizes only human TNF-α and chimpanzee; Etanercept binds and neutralizes human, cynomolgus, mouse TNF-α and human TNF-β; Certolizumab binds and neutralizes human and cynomolgus TNF-α, and Golimumab binds and neutralizes human, dog, cynomolgus, and rabbit TNF-α (Table 1B) (32–34).

**Table 1B:** Additional cross-reactivity information reported in a published assessment report^§^.

	**Human** ** (bind/neutralize)** ** B/N**	**Dog** ** (B/N)**	**Cynomolgus** ** (B/N)**	**Rat** ** (B/N)**	**Mouse** ** (B/N)**	**Rabbit** ** (B/N)**	**Pig** ** (B/N)**	**Human TNF-β** ** (B/N)**
Adalimumab (Humira®)	+/+	+/+	+/+	–/–	+/+	–/–	–/–	–/–
Infliximab (Remicade®)	+/+	–/–	–/–	–/–	–/–	–/–	–/–	–/–
Etanercept (Enbrel®)	+/+	–/–	+/+	n/a	+/+	n/a	n/a	+/+
Certolizumab (Cimzia®)	+/+	–/–	+/+	n/a	n/a	n/a	n/a	n/a
Golimumab (Simponi®)	+/+	+/+	+/+	–/–	–/–	+/+	n/a	–/–

§* European Medicines Agency Evaluation of Medicines for Human Use scientific report. +/– denotes yes or no binding/neutralization, respectively; n/a, data not available. This data does not include semi-quantitation of the degree of binding/neutralization seen in Table 1A*.

### *In vivo* Preclinical Safety of Quad-X™

**Figure 2:**
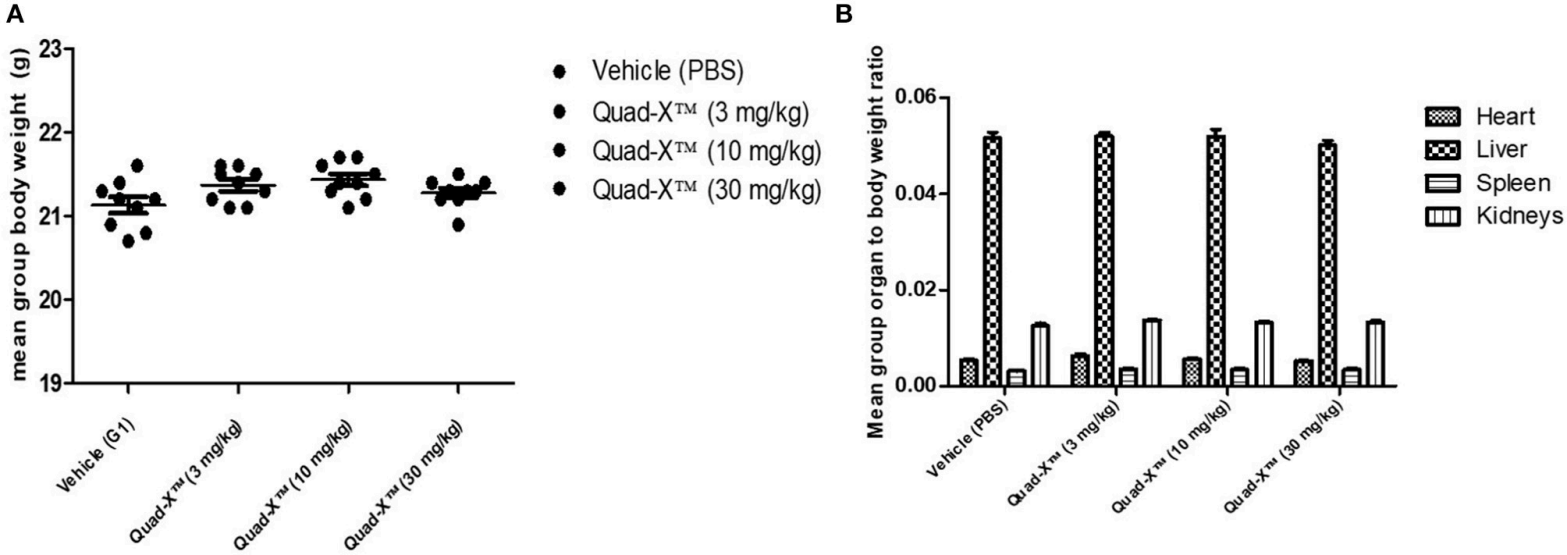
Preclinical toxicity assessment of Quad-X™ tested in C57BL/6 mice. **(A)** The effect of treatment on the mean body weight of experimental mice. The results shown are the means ± SEM (n = 10). Data was analyzed using an ANOVA multiple comparison test and Dunnett's post analysis (p-value > 0.1 in all group comparisons). **(B)** The effect of treatment on the mean organ to body weight ratio of experimental mice. Results shown are the means ± SEM (n = 10). Data was analyzed using the non-parametric Kruskal–Wallis multiple comparison tests with a Dunn's pair-wise test (p-value > 0.1 in all group comparisons vs. Vehicle treated group).

VNAR multivalent tandem and VNAR-Fc constructs have previously been successfully administered in several animal models including non-human primates, with no drug-associated toxicities reported (25, 26). However, this is the first time a Quad-X™ format (24) has been tested in an animal model and therefore, an indication of the maximum tolerated dose (MTD) in healthy mice was sought. C57BL/6 mice were treated with increasing doses of D1-Fc-C4 (Quad-X™) up to a maximum dose of 30 mg/kg administered subcutaneously and dosed twice within a week. These mice were monitored daily for 7 consecutive days for possible signs of any adverse treatment effects by recording animal viability, overall appearance, body function, behavior, body weight, water, and food intake. There were no observed adverse effects during the daily clinical observations and no significant body or organ weight changes observed between the control (PBS) and Quad-X™ treatment groups (Figure 2A). Food and water intake remained unchanged in the treatment groups compared to the untreated control group (data not shown). Following necropsy examinations, organs were found to be normal in colour, size, and consistency while cavities were clean with no signs of pathology. The ratio of the organ weights to body weights did not show any significant differences between different treatment groups (Figure 2B). The organ to body weight ratio is an assessment of possible abnormality and is indicative of a putative toxic effect of the test article.

### Efficacy of D1-BA11-C4 and Quad-X™ in Preventing Arthritis in Tg197 Mice

Anti-hTNF-α VNAR constructs, D1-BA11-C4 and D1-Fc-C4 (Quad-X™) which are potent (low pM) *in vitro* neutralizers of hTNF-α (24) were tested in the Tg197 transgenic human TNF-α model of polyarthritis, in which mice constitutively produce the pro-inflammatory human TNF-α cytokine, and consequently develop spontaneous arthritis. Two independent studies were conducted as described in Polyarthritis prevention in the Tg197 mouse model. In the first study, a standard preclinical dosing regimen of 3, 10, and 30 mg/kg was adopted as dosing at these levels has been well reported in this model (33, 35, 36). In the second study, the dosing regimen of our anti-hTNF-α constructs was titrated down to only 0.5, 1, and 3 mg/kg (study 1 showed complete disease control and no dose-dependent response, see below for details) vs. 1 and 3 mg/kg Humira®. For the first time in a clinically relevant animal model, a non-Fc based VNAR trimer construct (D1-BA11-C4) was administered parenterally at 30 mg/kg twice weekly for the duration of the study.

**Figure 3:**
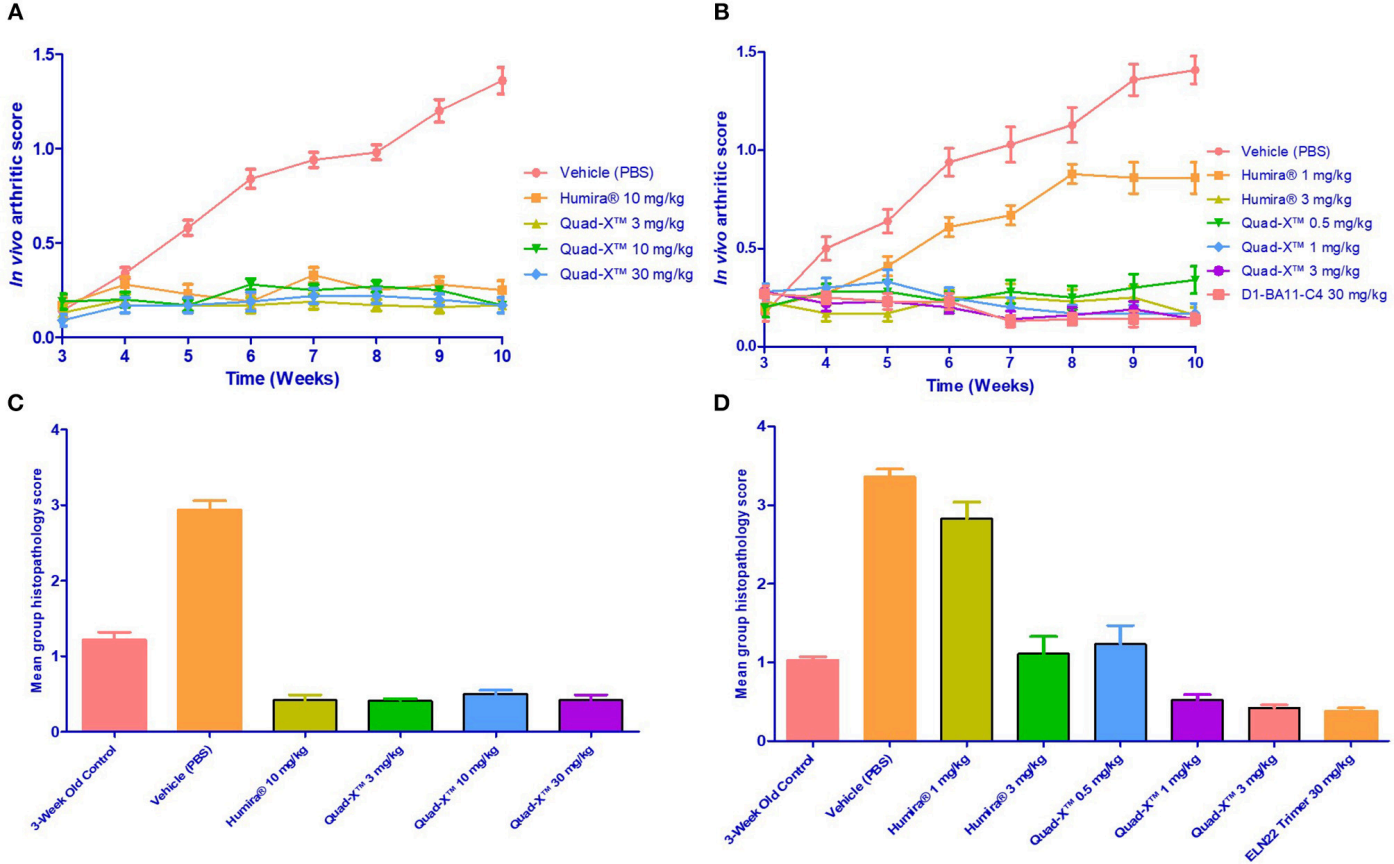
Efficacy of Quad-X™ and D1-BA11-C4 in preventing arthritis in Tg197 mice. **(A,B)** Mean *in vivo* disease severity scores of all groups treated twice weekly from week 3 of age. Results shown are the means ± SEM (n = 8). **(C,D)** Mean group arthritis histopathology scores at the end of the study (10 weeks of age). Results shown are the means ± SEM (n = 8). **(A,D)** Data were analyzed using the non-parametric Kruskal-Wallis multiple comparison tests with a Dunn's pair-wise test. **(A,B)** (p-value < 0.0005 in all group comparisons vs. Vehicle treated group, except for Humira® 1 mg/kg vs. Vehicle, p = 0.92). **(C,D)** (p-value < 0.0005 in all group comparisons vs. Vehicle treated group, except for Humira® 1 mg/kg vs. Vehicle, p = 0.1 and 3-week control group vs. Vehicle, p = 0.06).

**Figure 4:**
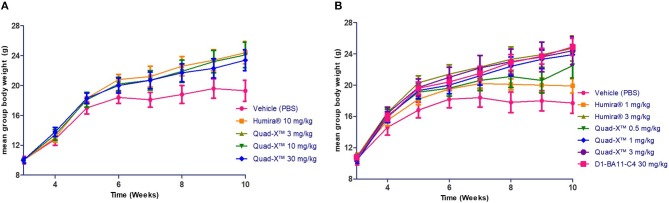
The effect of anti-hTNF-α treatment on the mean body weight of treated Tg197 mice. At the end of the study (10 weeks of age), the mean body weights of all groups treated twice weekly from week 3 were determined, and end of study weight scores obtained. Results shown are the means ± SEM (n = 8). Data was analyzed using an ANOVA multiple comparison test and Dunnett's post analysis. **(A)** (p-value < 0.05 in all group comparisons vs. Vehicle treated group). **(B)** (p-value < 0.05 in all group comparisons vs. Vehicle treated group except for Humira® 1 mg/kg vs. Vehicle, p = 0.77).

**Figure 5:**
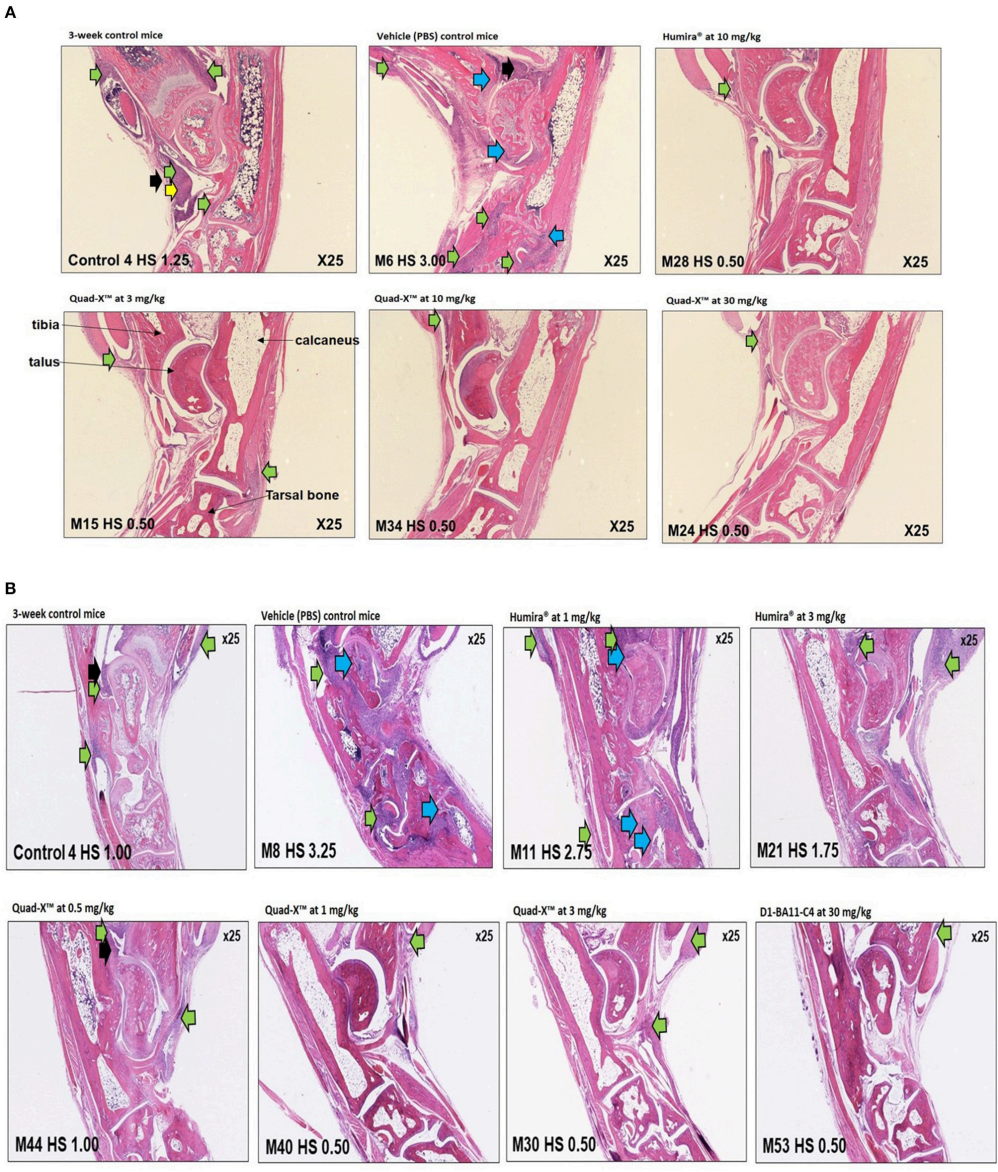
Representative histology images of ankle joints of treated Tg197 mice. Histology of inflammatory cell infiltration (green arrows), bone erosion (blue arrows), pannus (yellow arrows), synovial hyperplasia (black arrows), and significant healing of arthritic joints in young Tg197 mice. Tissue sections of the ankle joints were obtained from a 3-week-old baseline mouse grouping. In addition, tissue sections of the ankle joints were also obtained from a PBS treated vehicle control mouse group, anti-hTNF-α Quad-X™, D1-BA11-C4, and Humira® treated mouse groups at 10 weeks of age. Original magnification, 25x. **(A)** (From top, left to right: 3-week-control mice, vehicle (PBS) control mice, Humira® at 10 mg/kg; Bottom, left to right: Quad-X™ at 3, 10, and 30 mg/kg). **(B)** (From top, left to right: 3-week-control mice, vehicle (PBS) control mice, Humira® at 1 and 3 mg/kg; Bottom, left to right: Quad-X™ at 0.5, 1, and 3, D1-BA11-C4 at 30 mg/kg).

After 14 doses administered twice weekly for a period of 7 weeks, the results of the first study showed that all doses of Quad-X™ and D1-BA11-C4 at 30 mg/kg (second study) blocked the development of disease and sustained increase in body weight (Figures 3, 4, 5). *In vivo* arthritis and histopathology scores were inhibited by an average 88% and 86% respectively, across all anti-hTNF-α VNAR doses tested, with 0.5 mg/kg Quad-X™ inhibiting *in vivo* arthritis and histopathology scores at 76% (*p* = *0.0001*) and 63% (*p* < *0.0002*), 88% (*p* < *0.0001*) and 85% (*p* < *0.0001*) with 1 mg/kg Quad-X™, and 90% (*p* < *0.0001*) and 88% (*p* < *0.0001*) inhibition respectively with 3 mg/kg Quad-X™. Humira® inhibited arthritis and histopathology scores by an average of 84 and 76%, respectively at the 3 & 10 mg/kg dosing regimens, and the disease score inhibition at 1 mg/kg Humira® saw significant breakthrough of disease with values of only 39% (*p* = *0.923*) and 16% (*p* = *0.999*) for *in vivo* arthritis and histopathology scores, respectively. D1-BA11-C4 at 30 mg/kg dosing inhibited *in vivo* arthritis and histopathology scores by 90% (*p* < *0.0001*) and 89% (*p* < *0.0001*) respectively. The comparative examination of the arthritis inhibitory effects of Quad-X™ and Humira® at 1 mg/kg and 3 mg/kg dose level revealed that they were statistically undifferentiated in terms of their body weight gain measurement, but the *in vivo* arthritic and histopathological evaluations revealed that Quad-X™ displayed, with statistical significance, superior efficacy to Humira®. By, the end of study (week 10 of age) mean body weight for the 1 mg/kg Humira® treated mice group was 19.9 ± 0.9 g compared to 17.7 ± 1.3 g (PBS treated group) (*p* = *0.768*). Mean body weight in the 0.5 mg/kg and 1 mg/kg treated groups were 22.5 ± 1.6 g (*p* = *0.093*) and 23.9 ± 1.3 g (*p* = *0.017*) respectively. The average body weight of a healthy 10-week-old mouse is ≈ 25 g (male) and 20 g (female). The study 3-week-old control mice, week 3 mean group body weight was 7.3 ± 0.4 g. When translated into a human setting the effect of different doses on mean mouse body weight has been shown to correlate with both a decrease in joint pain levels and an increase in energy levels seen for RA patients.

A lower incidence of histopathology lesions at week 10 was observed in all Quad-X™ and D1-BA11-C4 treatment arms. These outcomes were statistically differentiated from the 3-week old control mice as well as the untreated (PBS treated) mice. Evidence of cell infiltration, bone erosion and cartilage degradation seen in 3-week-old and PBS-treated mice groups was present in 1 and 3 mg/kg Humira® treated mice (Figure 5).

The standard dosing regimen utilized in this *in vivo* efficacy study was based on the amount of drug in milligram per body weight of animal in kilograms (mg/kg), therefore did not correct for molecular size differences seen between formats (Humira®, 144 kDa, Quad-X™, 105 kDa and D1-BA11-C4, 40 kDa). In previously published *in vitro* efficacy experiments, an equimolar dosing regimen was adopted in an effort to correct for molecular size differences, with the VNAR Quad-X™ demonstrating 10x superior hTNF-α neutralizing potency when compared to Humira® (24).

## Discussion

About a decade ago, it was proposed that unlike VHH domains, VNARs might not efficiently form dimeric fusion molecules, and even where dimerization of VNAR was achieved, the resulting dimeric construct was published as having compromised expression levels and limited functional binding activity; postulated to be the result of steric hindrance or occlusion of the epitope by the incorporated linker (35, 37, 38). This speculation was later dismissed following the demonstration that VNARs tolerate both N- and C-terminal molecular fusions to form dimers and trimers without any obvious impact on their inherent biophysical properties or expressibility (39). Since these early reports, VNAR reformatting has become routine, and rational, extensive reformatting can generate tailor-made constructs specifically designed for either systemic, site-specific or topical delivery (24, 25, 40). These fusion constructs have been expressed in *E. coli*, yeast and mammalian cells at scalable yields and to the best of our knowledge, no evidence of significant aggregation of these domains, even when concentrated up to 150 mg/ml with prolonged storage, has been reported. Extending the half-life of therapeutic VNAR domains *via* molecular fusion is now achievable with a molecular mass of drug candidates at 25 kDa (dimer) or 40 kDa (trimer) using NDure™ (39, 40) or 80–100 kDa with an Fc fragment (24, 25). The benefits of smaller and simpler biologic drug formats cannot be over-emphasized and include: access to both extracellular and intracellular target antigens, non-systemic alternative routes of administration, reduced immunogenicity and cost/flexibility of production.

Although NDure™ fusion proteins and VNAR-Fc formats had been previously administered systemically in several preclinical proof-of-concept studies without *in vivo* toxicity [non-human primate (26), rat—manuscript in preparation, and mouse (25)], the Quad-X™ construct has never been *in vivo* before. This first example of a quadra-valent VNAR has demonstrated exceptional *in vitro* potency (2–5 picomolar) when compared with conventional anti-TNF-α biologics in the clinic (24, 41–43). In a preliminary MTD study in C57BL/6 mice, the safety of the anti-TNF-α Quad-X™ at 30 mg/kg, administered subcutaneously, was confirmed. There was no clinical evidence of acute or delayed adverse events in these treated mice.

With its unique and protruding binding loops (CDR1 and CDR3), we speculated that these anti-TNF-α domains (VNAR D1 and C4), forming the backbone of the D1-BA11-C4 and D1-Fc-C4 Quad-X™ constructs, may be interacting with unique and previously unseen “recessed” epitopes on the TNF-α molecule, in stark contrast to the epitopes recognized by the planar paratopes of the commercially available biologics including even VHH (35, 44–47). In a comprehensive TNF-α species cross-reactivity characterization study, the lead anti-TNF-α VNARs appeared to bind a unique TNF-α epitope. In addition, and using data collected from the European Medicines Agency Evaluation of Medicines for Human Use scientific report detailing other commercially available TNF-α and TNFR1 antagonist biologics (Tables 1A,B), whilst the binding/neutralization profile of our VNAR domains share some similarity with Humira® and Simponi®, these two biologics also bind/neutralize mouse and rabbit TNF-α, respectively. In a BIAcore™ epitope binning study, a VNAR D1-C4 dimer and VHH TNF30-TNF30 recognized different epitopes on immobilized human TNF-α (data not shown). Although for cost effective drug development, it is beneficial to demonstrate broad species cross-reactivity, facilitating proof-of-concept preclinical studies in inexpensive animal models, restricted cross-reactivity seems to be a feature of improved human efficacy of TNF-α protein antagonist (32–34). In Table 1, there is a striking and restricted cross-reactivity across all listed clinical biologics. This restricted cross-reactivity is no longer considered a limitation to the development of anti-TNF-α biologics as the use of surrogate domains and transgenic animal models of human disease (expressing human TNF-α) is now a well-worn and successful clinical path (32, 48–50). In parallel to the work published here, we have solved the structure of the anti-TNF-α VNAR-human TNF-α complexes and the analysis of this crystallization data has confirmed the binding of VNAR to a novel epitope. Like Humira® (47), one VNAR molecule interacts with two adjacent TNF-α protomers. However, this is where the similarity ends with only 10% of the TNF-α contact residues common to both proteins (manuscript in preparation).

It is sometimes easy to forget that the original regulatory and clinical path of anti-TNF biologics required the use of surrogate binders in poorly predictive anti-inflammatory animal models. This enforced strategy was adopted because molecules such as Humira^Ⓡ^ (and here the Quad X™) are unable to bind and neutralize, with any therapeutically relevant potency, rodent TNF-α. It is only with the recent development of new transgenic disease models (27) that interrogation of a system that approaches that seen in man is now possible. Although there is strong evidence that models such as Tg197 mice are highly predictive of future efficacy in man, these *in vivo* pre-clinical data sets are typically less comprehensive than those generated for other indications such as cancer, where the use of multiple, related *in vivo* models is often possible.

The development of TNF-α transgenic mice (Tg197) carrying a 3'-modified UTR human TNF-α gene construct allows deregulated soluble human TNF-α gene expression *in vivo*. These mice develop chronic inflammatory polyarthritis resembling human rheumatoid arthritis as early as 4 weeks after birth (27, 51, 52). It is clear that all types of RA and IBD in (human) patients don't begin with dysregulated TNF expression and certainly not with the rapidity of the disease progression seen in Tg197 mice. However, this model has proved to be an invaluable *in vivo* tool capable of accurately forecasting the therapeutic potency of anti-TNF drugs and accelerating the development of next-generation anti-TNF-α biologic therapies. Where they have been less informative is their ability to predict some of the now well-recognized side effects that are associated with longer term anti-TNF-α therapies such as increased susceptibility to opportunistic infections, malignancies and cardiovascular risks (53–56). While inflammatory disease in the Tg197 mouse model is solely driven by soluble TNF-α, it is worth emphasizing that the soluble TNF-α precursor, transmembrane TNF-α (tmTNF-α), is not only a mediator of immunity but also displays anti-inflammatory and anti-apoptotic reactions (57–59). Whilst establishing the tmTNF-α binding and neutralizing profile of our VNARs may potentially add value to our current knowledge, here it is the clinical importance of targeting soluble TNF-α that has been the focus of these studies, since it is binding and neutralization of the soluble form of TNF-α that has been shown to be the critical and common mechanism of action of all approved anti-TNF-α therapeutic agents (60).

The *in vivo* efficacy of Quad-X™ and D1-BA11-C4 in a Tg197 transgenic human TNF-α polyarthritis disease model reinforces the validity of our previously reported potent *in vitro* neutralization data which saw an order of magnitude improvement over Humira® (24). In this present study, clinical efficacy seen with a 1 mg/kg dosing of Quad-X™ is slightly better than, but comparable to, that observed with a 10 mg/kg Humira® dose. In addition, breakthrough of underlying disease was observed for both the 1 and 3 mg/kg Humira® treatment regimens. It has previously been established that a 3 mg/kg Humira® dosing is sub-therapeutic, at least in this disease model (36). Although the Quad-X™ format clearly demonstrated improved efficacy in this transgenic model over Humira®, it would be premature to make assumptions about reducing any longer-term risks such as greater susceptibility to infection in the absence of clinical data. The trimer construct D1-BA11-C4 (40 kDa), is a second rationally designed therapeutic format incorporating on this occasion an anti-HSA binding domain, NDure™ (which cross-reacts with mouse serum albumin) to extend serum half-life close to that of a wild type immunoglobulin Fc region (39). The VNAR BA11 domain located in the middle of the two anti-TNF-α also functions as a spacer alongside the flexible 20 amino acid Gly_4_Ser linker flanking both N- and C- terminals of BA11. The presence of a spacer has previously been associated with a significant enhancement in *in vitro* potency (24), and is an effect that has been reported by other independent researchers (35, 61). This relatively low molecular weight, super-potent trimer may also be well-suited to topical and/or site-specific delivery in autoimmune conditions such as IBD, uveitis, and psoriasis. A combination of topical and site-specific delivery and the absence of an Fc region is typically associated with reduced systemic toxicity (35, 62, 63), further extends the format's utility and compliance.

VNAR domains are evolutionarily distant from human and other lower warm-blooded mammals, but evidence from both *in silico* and dendritic cell-T-cell assays, capable of identifying the presence or absence of potential T-cell epitopes, support their inherent non-immunogenic features, at least in humans (40), with an established route to humanization confirmed for a number of VNAR domains (39). A humanized VNAR domain, such as NDure™, is often referred to in the literature as a soloMER™ (24, 40, 64).

## Conclusion

While dose escalation and in-class switching are approaches utilized by clinicians to manage most anti-TNF-α biologics non-responder patients, dose escalation eventually leads to increased treatment cost, increased systemic toxicity, accelerated immunogenicity, infusion reaction, and eventual termination of therapy (13, 16, 65, 66). The anti-TNF-α Quad-X™, which is about two-thirds of the size of a conventional mAb, and the anti- TNF-α NDure™ fusion product (40 kDa) both clearly have the potential to be new and useful additions to the existing formulary of anti-TNF biologics available to clinicians. Where these drug candidates show real advantage as future disruptive therapies, is through their enhanced *in vivo* potency and controlled PK/delivery, predicting their use at significantly lower doses than current standard of care biologics. It is hoped that this tailoring of drug use will, in turn, limit the risk of anti-drug antibodies, control/optimize the costs of drug therapy and begin to reduce the systemic toxicity risks [infection and malignancies (53–55, 67, 68)] seen from the practice of extended dose escalation therapies.

## Ethics Statement

All reported animal experiment was conducted at the Biomedcode SA test facility. All procedures conformed to the Presidential Decree No 56/2013 Government Gazette No A' 106 applicable in Greece (EEC Directive 2010/63/ECC) and were approved by the directorate of Agricultural and Veterinary Policy (DAVP) of the Attica Region. Approved license Protocol No. 3798/3-07-2017.

## Author Contributions

OU, scientific lead on this paper, conducted the majority of the scientific work and wrote the manuscript. JS assisted in the rational *in-silico* design and preliminary expression of all anti-TNF VNAR constructs described in this publication. CB led the science team at Elasmogen Limited and co-supervised this project alongside AP. Also, CB and AP reviewed the manuscript.

### Conflict of Interest Statement

Authors affiliations are clearly stated including those affiliated with the biotechnology company, Elasmogen Limited.
